# Talking Less during Social Interactions Predicts Enjoyment: A Mobile Sensing Pilot Study

**DOI:** 10.1371/journal.pone.0158834

**Published:** 2016-07-20

**Authors:** Gillian M. Sandstrom, Vincent Wen-Sheng Tseng, Jean Costa, Fabian Okeke, Tanzeem Choudhury, Elizabeth W. Dunn

**Affiliations:** 1 Department of Psychology, University of British Columbia, Vancouver, B.C., Canada; 2 Department of Information Science, Cornell University, Ithaca, N.Y., United States of America; University of Vermont, UNITED STATES

## Abstract

Can we predict which conversations are enjoyable without hearing the words that are spoken? A total of 36 participants used a mobile app, My Social Ties, which collected data about 473 conversations that the participants engaged in as they went about their daily lives. We tested whether conversational properties (conversation length, rate of turn taking, proportion of speaking time) and acoustical properties (volume, pitch) could predict enjoyment of a conversation. Surprisingly, people enjoyed their conversations more when they spoke a smaller proportion of the time. This pilot study demonstrates how conversational properties of social interactions can predict psychologically meaningful outcomes, such as how much a person enjoys the conversation. It also illustrates how mobile phones can provide a window into everyday social experiences and well-being.

## Introduction

People generally enjoy talking to one another. When asked how they are currently feeling, people report being happier during social activities/interactions than during non-social activities [1]. When people think back on their day, they remember being happier during times in which they were socializing than during times in which they were doing other activities [2,3]. Further, people report being happier on days in which they recall more social activities [4–7]. These effects extend not only to interactions with close others, but also to interactions with people who are more peripheral in our social networks: people report being happier on days when they interact with more close friends and family, but also on days when they interact with more acquaintances [8].

Although, in general, the more social interactions a person has, the happier they feel, this conclusion ignores the fact that interactions differ in quality: Not every interaction results in equally positive feelings. Conversations that involve receiving help or support, and conversations that involve arguing or confrontation are associated with increases in negative affect [6]. In contrast, the more enjoyable a conversation is, the more positive affect a person feels after the conversation [4]. These findings suggest that the quality of a conversation is related to the emotional response to that conversation. In turn, these emotional responses have implications for well-being, especially for older adults [9,10]. Given the difficulty of tracking every conversation that a person has, and the burden of reporting on the emotional response to each conversation, past research has often relied on retrospective, aggregate reports. Could there be another way to assess conversation quality?

The emotional quality of a conversation is not simply a function of what is said, but also *how* it is said. Research on the communication of emotion in music highlights the importance of prosody: linguistic features such as intonation, tone, stress and rhythm [11,12]. Auditory signals of pitch and loudness can be assessed through acoustical information (e.g., fundamental frequency and intensity [13]). Individual acoustical features (e.g., pitch, volume, and speech rate) have been linked to judgments of psychological constructs, such as power/dominance and competence. When men lower the pitch of their voice, others attribute higher social dominance [14–16]. Pitch also affects judgments of competence. In a forced choice design, male targets with lower pitched voices were judged to be significantly more competent (better leaders, more intelligent) than targets with higher pitched voices [16]. Volume is another acoustical feature associated with judgments of power/dominance; people associate trait dominance with loud voices [17]. Although these judgments could be merely a result of unfounded stereotypes, in fact people are capable of making relatively accurate judgments of others’ personalities if they hear, but don’t see the person [18].

The emotional quality of a social interaction may be related to not only acoustical features, but also structural features of the conversation. Computer scientists in the emerging area of social signal processing posit that computers can be empowered with the ability to sense and understand human social signals [19]. Experiments seeking automated ways to detect social signals have found that speaking time and interruptions are related to dominance [20–22], and that turn-taking patterns are related to social influence [23,24]. Turn-taking, as a measure of engagement, is also related to liking (e.g., in one study: feelings towards a speed-dating partner [25]).

Taken together, past research suggests that the acoustical and conversational properties of a social interaction might be related to psychological outcomes, such as emotional responses. Mobile phones provide an ideal means of capturing both kinds of properties because they are portable and are equipped with a wide array of unobtrusive sensors. The microphones built into mobile phones can pick up on in-person conversations even when the phone is not in use, providing an acoustical trace from which conversational and acoustical properties can be extracted.

Further, by using a phone app, this acoustical information can be collected unobtrusively, providing a window into real-world, everyday social experiences instead of into artificial social experiences created in a laboratory. All of this can be done while maintaining the privacy of both conversation partners; the auditory signal can be pre-processed on the phone so that only information like volume and pitch is sent to the researchers (i.e., formant information is not preserved, so that no raw acoustical data, such as voices/words, leave the phone [26]).

We used a mobile phone app, My Social Ties, to capture information about the social interactions people had as they went about their daily lives. In this pilot study, we explored whether the conversational and acoustical properties we extracted from a social interaction could predict the emotional response to the interaction. In essence, we wanted to know whether we could predict which conversations were enjoyable without hearing the words that were spoken.

## Methods

### Participants

We recruited 60 undergraduate students with Android phones, who participated in exchange for class credit or $30. One student was removed from the study due to non-compliance and 4 students withdrew from the study because they were unhappy with the app (the audio files took up a lot of space on their phones and the app depleted their phone’s battery). Due to technical difficulties related to downloading the enormous audio files from students’ diverse phones (which included Samsung, HTC, LG, Dell, Motorola, and Sony), we had no acoustical information for 2 students. Due to file corruption, we could not process the acoustical information for an additional 9 students. These kinds of technical issues are not unexpected with non-commercial apps that are developed for small-scale use. Finally, given that hierarchical linear modelling has a practical minimum of three data points per person, we dropped 8 participants who had fewer than three conversations each, leaving us with a sample of 36 participants (21 females, 14 males, 1 did not report their sex; *M*_age_ = 20.6, *SD*_age_ = 4.92). These participants had a total of 473 conversations (range = 3 to 58; *M* = 13, *SD* = 12).

### Procedure

This research involving human participants was approved by the Behavioural Research Ethics Board of the University of British Columbia [H12-00469]. Participants came to the lab and provided written consent. Participants then filled out a survey with demographic information (including sex and age), and completed an abbreviated 21-item version of the Big Five Inventory measuring Openness, Conscientiousness, Extraversion, Agreeableness, and Neuroticism [27], plus three perceived intelligence items (for exact items see [28]).

Next, research assistants explained that the study involved installing an app, My Social Ties, on their Android phones, which they would use for 6 days. (NOTE: My Social Ties is not publically available, but if you are interested in the possibility of using it for research purposes, please contact tanzeem.choudhury@cornell.edu.) The research assistants explained that the app would store audio data collected during participants’ conversations, but not any raw audio (i.e., their voice and conversation content could never be heard). The app was then installed on each participant’s phone. Each participant read part of a story out loud to provide training data for the app, then sat in silence for one minute so that the app could detect the end of the conversation (i.e., the participant reading the story to the research assistant). Upon detecting the end of a conversation, the app triggered a survey, which asked participants to rate how they had felt during the conversation (1 = *very unhappy*, 7 = *very happy; M* = 4.31, *SD* = 1.25) (see [29] for the full list of questions on the momentary survey).

The audio files collected from participants’ phones were parsed through a two-step process. First, we identified the voiced segments of the conversation and eliminated potential noises from the environment by using a method that has been validated on privacy-sensitive audio information [26]. Second, we performed speaker diarization, to identify the voiced segments where the participant was speaking, where other people were speaking, and where there was non-speaking noise and silence. Given that we did not retain raw audio data, we could not do speaker diarization manually. Instead, we used k-means clustering (with a random seed) [30] to break each individual conversation into segments based on volume (i.e., energy intensity). Previous studies have shown that k-means clustering is capable of achieving good results on conversations containing any number of speakers [31,32]. Since we were interested in the data of only one speaker (the person using the My Social Ties app), we were able to use a fixed set of clusters (k = 4): 1) Extremely high (i.e., might not be heard by human ears)—noise of the phone rubbing against clothing; 2) High—Voice of the person closest to the phone (i.e., the participant); 3) Low—Voices of other people; 4) Extremely low—Silence. Our first attempt at diarization resulted in an artificially high number of segments, as a result of being overly sensitive to the ups and downs and pauses within the volume fluctuations of a single speaker (e.g., misclassifying a participant’s lowest volume conversation segments as belonging instead to their conversation partner; see [33]). Consequently, we ran a smoothing algorithm that assumed a minimum speaking time of 1.5 seconds. Finally, we removed the chunk of silence at the end of each conversation that was needed for the app to determine whether or not the conversation was terminated.

The acoustical and conversational properties of interest were extracted or computed based on the output of the diarization process (i.e., information about which voiced segments corresponded to the participant speaking, and which corresponded to someone else speaking). The volume and pitch were extracted from each voiced segment and we computed an average (min_vol_ = 39 db, max_vol_ = 65 db; *M*_vol_ = 57 db, *SD*_*vol*_ = 5 db; min_pitch_ = 92 Hz, max_pitch_ = 253 Hz; *M*_pitch_ = 182 Hz, *SD*_pitch_ = 36 Hz) and standard deviation (*M*_vol_ = 53 db, *SD*_*vol*_ = 5 db; *M*_pitch_ = 39 Hz, *SD*_pitch_ = 23 Hz) over all the voiced segments that corresponded to the participant speaking. The conversation length (min = 30 sec, max = 128 min; *M* = 8.6 min, *SD* = 15.5 min) indicates the length of the entire audio file (which corresponds to a detected conversation), except for the chunk of silence at the end. The percentage of time that the participant was speaking (min = 1%, max = 95%; *M* = 40%, *SD* = 22%) indicates the total amount of time that the participant spoke, divided by the conversation length. Finally, the rate of turn-taking (min = 1 per min, max = 18 per min; *M* = 7 per min, *SD* = 4 per min) indicates how many times people took turns speaking per minute (i.e., the number of voiced segments from the diarization process). For example, if the participant spoke, then someone else, and then the participant again, that would represent 3 turns.

An additional 65 conversations, not included in the descriptives, were discarded because of possible corruption or inaccurate diarization: the computed speaking time was less than 0 (*N* = 9), there was no time when the participant was not speaking (*N* = 14), the rate of turn-taking was abnormally high (more than 3 SD’s above the mean; *N* = 6), the average volume was abnormally high (more than 3 SD’s above the mean; *N* = 8), or the average pitch was higher than the maximum of the typical adult range (i.e., an average greater than 255 Hz; *N* = 28). As mentioned earlier, these kinds of technical issues are not unexpected with non-commercial apps that are developed for small-scale use.

## Results

The conversational properties were marginally related to one another: conversation length was significantly correlated with percentage of time spent speaking, *r*(471) = -.29, *p* < .001, but not with rate of turn-taking, *r*(471) = -.02, *p* = .66. Percentage of time spent speaking was not significantly correlated with rate of turn-taking, *r*(471) = -.07, *p* = .11. As to the acoustical properties, average volume was significantly correlated with average pitch, *r*(471) = -.37, *p* < .001, and variability in volume was significantly correlated with variability in pitch, *r*(471) = .19, *p* < .001.

Given the extremely large correlation between average volume and variability in volume, *r*(471) = .90, *p* < .001, and the consequent likelihood of multicollinearity, it was important to use either average or variability in the subsequent analyses, but not both. Given that past research has focussed on variability [34], we used variability in volume and pitch as predictors in our analyses.

We capitalized on the fact that each person had multiple conversations by running within-person analyses using hierarchical linear modelling (HLM) via the lme4 package in R [35], with conversation as the Level 1 variable, and person as the Level 2 variable. We predicted the emotional response to a conversation from conversation length, percentage of time spent speaking, rate of turn-taking, and variability in volume and pitch (all z-scored and entered simultaneously). Given that we lacked specific predictions, all analyses should be considered exploratory.

Conversations during which a person spent a smaller percentage of time speaking were enjoyed more, β = -.19, *t*(30) = -3.98, *p* < .001. In contrast, neither the conversation length, β = -.002, *t*(30) = -0.04, *p* = .98, nor the rate of turn-taking, β = -.001, *t*(30) = -0.03, *p* = .98, predicted how one felt after a conversation. Neither variations in volume β = -.05, *t*(30) = -0.88, *p* = .39, nor variations in pitch, β = .05, *t*(30) = 1.09, *p* = .28, predicted enjoyment.

Although our study is under-powered to test individual difference variables, and although individual differences were not the focus of our study, we examined the extent to which individual differences could predict conversation enjoyment. When all of the big-five personality traits, age, and gender were added to the model, none of these individual differences significantly predicted feelings, β’s < .10, *p*’s > .39.

We also ran exploratory analyses to test for relationships between the acoustical and conversational properties (averaged across participants) and the individual difference variables (personality, age, gender). Neither average volume nor variability in volume was significantly correlated with any individual difference variables, *r*’s < .26, *p*’s > .14, and did not differ by gender. As expected, average pitch was higher for women than for men, *t*(33) = 3.18, *p* = .003. Additionally, older participants spoke with a somewhat lower average pitch than younger participants, *r*(33) = -.30, *p* = .08. Pitch was not significantly correlated with any other individual difference variables, *r*’s < .21, *p*’s > .24. Variability in pitch was not significantly correlated with any individual difference variables, *r*’s < .17, *p*’s > .33, and did not differ by gender. Conversation length was not significantly correlated with age or personality, *r*’s < .23, *p*’s > .19, but men had somewhat longer conversations than women *t*(33) = 1.99, *p* = .06. The percentage of time spent speaking was marginally higher for older people, *r*(33) = .29, *p* = .09, and, surprisingly, marginally lower for extraverted people, *r*(33) = -.30, *p* = .08, but was not significantly correlated with any other individual difference variables, *r*’s < .28, *p*’s > .11, and did not differ by gender. Finally, rate of turn-taking was not significantly correlated with any individual difference variables, *r*’s < .22, *p*’s > .21, and did not differ by gender.

## Discussion

We used a mobile phone app to unobtrusively gather acoustical information about conversations that people had in their everyday lives. People’s enjoyment of a social interaction can be predicted from conversational properties of that interaction. People enjoyed their conversations more when they spoke a smaller proportion of the time than usual. These effects were not moderated by personality, age, or gender.

Although our findings are based on only 36 people, those people had 473 conversations. Thus, the use of a more powerful within-person design bolsters the conclusions despite the small sample size. Given that the data for this study were collected via a mobile phone app, and given that mobile phone apps can be easily distributed via online app stores, future studies have the potential to collect large amounts of data from geographically distributed people who download and install the app on their own.

Future studies are needed to establish the generalizability of these findings. Indeed, several factors could moderate the relationship between acoustical/conversational properties and enjoyment. In a past study by Yuan and colleagues [36], speaking rate was found to vary by gender, age, and conversation partner: females, older people, and conversations with strangers tend to exhibit slower speaking rates. Although we didn’t ask participants specifically about conversations with strangers, we did ask whether each conversation was with a strong tie (e.g., close friends and family), a weak tie (e.g., acquaintances), or someone else. When we looked solely at the conversations with strong ties (*N* = 210) and weak ties (*N* = 197), we found no difference in how much participants enjoyed their conversations, and there were no differences in any of the acoustical (variability in volume, variability in pitch) or conversational features (conversation length, rate of turn-taking, or percentage of time spent speaking) depending on the conversation partner.

Culture is another possible moderator of the relationship between acoustical/conversational properties and enjoyment. On the predictor side of the equation, there is some evidence that women in various cultures exhibit differences in average pitch [15]. This might suggest that future replication efforts should focus on a single culture, and that the effect should be tested in several cultures that are known to vary in average pitch. On the outcome side of the equation, cultural differences in the extent to which people rely on internal speech result in differences in performance on reasoning tasks. It is not implausible that these differences might also have affective consequences, manifesting in differences in enjoyment.

At face value, the finding that talking a smaller proportion of the time resulted in more enjoyable conversations seems at odds with the fact that people who are depressed tend to talk less; the Center for Epidemiologic Studies’ Depression Scale (CES-D) includes an item “I talked less than usual [during the past week]” [37]. However, we suspect that this item refers to the number of social interactions a person engages in, rather than the amount of talking during each social interaction. This interpretation is consistent with the finding that people are happier on days when they have more social interactions, whether with close friends and family or with acquaintances [8]. Future studies should further examine the distinction between these two constructs (i.e., amount of talking within a conversation vs. number of conversations). Also, it remains to be seen whether there is a minimum amount of talking within a conversation that yields benefits; in our experience, a conversation where you can never get a word in edgewise is not too enjoyable.

The current work has implications for Pentland’s [34] theory of social signals. Our finding, that we can predict the emotional response to a social interaction from its conversational properties, is consistent with Pentland’s idea that acoustical properties of interactions act as social signals. Pentland describes how to measure four types of social signals: activity level (proportion of time a person is speaking), engagement (the extent to which one person’s turn-taking is influenced by the other’s), stress/emphasis (variation in pitch and volume), and mirroring (mimicking the other’s short utterances). The features that we examined map on quite closely to the features that he proposed: our proportion of speaking time maps onto his activity feature, our rate of turn-taking is similar to his engagement feature, and our variation in volume and pitch are analogous to his stress/emphasis feature. However, we could not analyze a feature that is similar to his mirroring feature, since participants’ conversation partners generally did not have our mobile app, My Social Ties, installed on their phones.

Indeed, one limitation of this study is that we did not report any acoustical features related to the conversation partner. Intuitively this is important, as each conversation partner will influence the other, and both parties seem likely to influence the emotional response to a conversation. Future studies could examine not only the volume and pitch of the conversation partner, but also measure synchronicity of volume and pitch.

Another limitation of this study is that the results are dependent on knowing when a participant is speaking during each conversation, and when they are not speaking (i.e., dependent on accurate speaker segmentation and diarization). We were unable to do these steps manually, due to the privacy-sensitive app that we used. Instead, we used automated methods that have been validated against manual methods [26, 31], but no automated method is 100% accurate. Although inaccuracies are inevitable, we have no reason to believe that these inaccuracies would result in a spurious relationship between the enjoyment of a conversation and the percentage of speaking time (but not other conversational or acoustical properties).

The unobtrusive, privacy-maintaining method used in the current study shows vast potential as a tool for psychological study. With more than 2.5 billion people around the world already carrying around smartphones as they go about their daily lives [38], there is a huge opportunity to harness mobile apps for the psychological study of everyday behavior. Physicians and clinicians could use a mobile app to monitor patients who have difficulty communicating (e.g., people with social anxiety disorder, or people with Parkinson’s disease; [39]), and use the data to potentially feed into treatment plans. Psychologists could use a mobile app to understand the ways in which people interact differently with outgroup members, to test whether an intervention (e.g., the “fast friends” procedure [40]) changes the way people interact with others, or to test myriad other questions.

This pilot study demonstrates how the conversational and acoustical properties of social interactions can predict psychologically meaningful outcomes, such as how much a person enjoys the conversation. In other words, even without hearing the content of a conversation, we can predict the emotional response to it. The current work also illustrates the potential of mobile sensing to provide a window into everyday social experiences and well-being.
